# Childhood overweight and obesity and the risk of depression across the lifespan

**DOI:** 10.1186/s12887-020-1930-8

**Published:** 2020-01-21

**Authors:** Deborah Gibson-Smith, Thorhallur I. Halldorsson, Mariska Bot, Ingeborg A. Brouwer, Marjolein Visser, Inga Thorsdottir, Bryndis E. Birgisdottir, Vilmundur Gudnason, Gudny Eiriksdottir, Lenore J. Launer, Tamara B. Harris, Ingibjorg Gunnarsdottir

**Affiliations:** 1 Department of Health Sciences, University of York, York, UK; 2 Unit for Nutrition Research, Landspitali, The National University Hospital of Iceland and Faculty of Food Science and Nutrition, School of Health Sciences, University of Iceland, Eiriksgata 29, 101, Reykjavik, Iceland; 3 Department of Epidemiology Research, Centre for Fetal Programming, Statens Serum Institut, 5, Artillerivej, 2300 Copenhagen S, Denmark; 4 Department of Psychiatry, Amsterdam Public Health Research Institute, Amsterdam UMC, Amsterdam, The Netherlands; 5 Department of Health Sciences, Faculty of Science and Amsterdam Public Health research institute, Vrije Universiteit Amsterdam, the Netherlands, Vrije Universiteit Amsterdam, 1081 HV Amsterdam, The Netherlands; 6 Department of Internal Medicine, Nutrition and Dietetics, VU University Medical Center, Amsterdam, The Netherlands; 7 Icelandic Heart Association, Holtasmari 1, 201, Kopavogur, Iceland; 8 National Institute on Aging, Laboratory of Epidemiology, and Population Sciences, 7201 Wisconsin Avenue, Bethesda, MD 20892-9205 USA

**Keywords:** Childhood obesity, Depressive symptoms, Lifetime major depressive disorder, Body mass index

## Abstract

**Background:**

Obesity has been longitudinally associated with depression but only few studies take a life course approach. This longitudinal study investigates whether being overweight or obese at age 8 and 13 years is associated with depressive symptoms more than 60 years later and whether this association is independent of late-life body mass index (BMI). We also investigated the association of being overweight/obese at age 8 or 13 years with ever having major depressive disorder (lifetime MDD).

**Method:**

This analysis is based on a sub-sample of 889 AGES-Reykjavik participants with measured BMI data from early life. Late-life depressive symptoms were measured with the Geriatric Depression Scale (GDS) and lifetime MDD was assessed at late-life using the Mini International Neuropsychiatric Interview. Logistic regression analysis was used to estimate the relationships between BMI (continuous and categorical) at age 8 or 13 years, and late-life depressive symptoms (measured as GDS ≥ 5) or lifetime MDD, adjusted for sex, education, physical activity, smoking status and alcohol use. In a separate model, additional adjustments were made for late-life BMI.

**Results:**

One hundred and one subjects (11%) had depressive symptoms at late-life (GDS ≥ 5), and 39 subjects (4.4%) had lifetime MDD. Being overweight or obese at age 8 or 13 years was not associated with higher depressive symptoms during late-life, irrespective of late-life BMI. Being overweight or obese at age 8 years, but not age 13 years was associated with an increased risk of lifetime MDD (Odds Ratio (OR) (95% confidence interval [CI]) for age 8 = 4.03[1.16–13.96]*P* = 0.03 and age 13 = 2.65[0.69–10.26] *P* = 0.16, respectively).

**Conclusion:**

Being overweight in childhood was associated with increased odds of lifetime MDD, although the magnitude of the risk is uncertain given the small numbers of participants with lifetime MDD. No clear association was observed between childhood and adolescent overweight/obesity and late-life depressive symptoms irrespective of late life BMI.

## Introduction

The prevalence of children with overweight and obesity is increasing. In developed countries, the age-standardized prevalence in children and adolescents (ages 2–19 years) has increased from 16.9% in 1980 to 23.8% in 2013 for boys and 16.2 to 22.6% for girls [1]. Studies which take a life course approach have suggested that early-life obesity can lead to poorer later-life health outcomes including an increased risk for cancer [2], diabetes [3], hypertension [4] and cardiovascular disease mortality [5]. Hence having obesity during childhood is a potential risk factor for adult morbidity. Several studies have focused on the link between adolescent with obesity and mental health during early or middle adult life [6–9] as adolescence is an important developmental period where appearances and peer approval are key values. Increased body dissatisfaction, low self-esteem and perceived stigmatization due to obesity are hypothesized to increase the risk of psychiatric disorders and in particular, depression [10, 11]. An alternative explanation is a shared genetic risk, which has been suggested as a factor linking obesity and depression [12].

Despite several studies [6, 8, 9, 13] examining the relationship between adolescent with obesity and depression uncertainties remain, such as whether the relationship is age-dependent. Studies examining children with obesity (obesity under the age of 12y) and depression at adolescence and adulthood have found inconsistent results (9, 14,-16). For example, two studies found that childhood overweight/obesity is associated with an increased risk of mood disorders in adulthood [9, 14], while others found no association [15] or found inconsistent associations at different childhood ages [16]. Furthermore, few studies have a long enough follow-up to study the association between childhood obesity with depression over the whole life course. The influence of having overweight in early-life on late-life mental health was investigated by Martinson et al.(2016) who found that adolescent girls with overweight (but not boys) had a 1.74 greater odds of experiencing depression symptoms at age 65 than their normal weight counterparts [6]. This study was limited by the use of estimated body weight extrapolated from high school photographs. Additionally, this and many other studies did not consider that having overweight during childhood is largely predictive of having overweight and obese during adulthood [17]. How much the observed relationship between early-life weight and later-life depression is explained by obesity at later life is currently unknown.

The purpose of this study was to investigate the association between measured body mass index (BMI) in childhood/ early adolescence and its relationship with depression over a lifetime. The significance of this study is its long-term follow-up enabling us to adopt a life-course approach to weight and depression. The following questions were addressed [1] Is BMI in childhood (age 8 years) and adolescence (age 13 years) related to late-life depressive symptoms (measured at age ~ 75y)? [2] Is BMI in childhood and adolescence related to late-life depressive symptoms irrespective of late-life BMI? [3] Is BMI in childhood and adolescence related to lifetime MDD (~ 65 years follow-up)?

## Method

### Study population

**Fig. 1:**
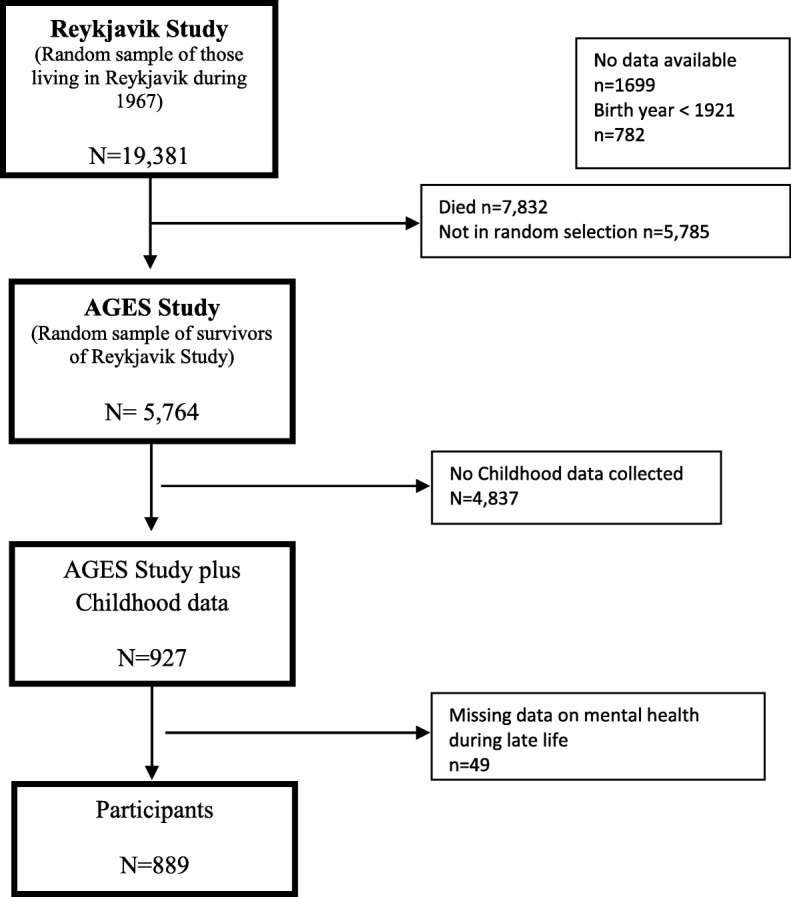
Flow diagram of data collection

The AGES-Reykjavik (Age, Gene/Environment Susceptibility) cohort is drawn from a random selection of survivors from the established population-based cohort, the Reykjavik Study (1967–1991) (*n* = 19,381). The Reykjavik study is a cohort of men and women born between 1907 and 1935 that has been followed in Iceland since 1967 by the Icelandic Heart Association. The AGES-Reykjavik study was a follow-up study designed to examine risk factors, including genetic susceptibility and gene/environment interaction, in relation to disease and disability in old age. Data measurements were performed during 2002–2006 and included blood draws, electrocardiograms, anthropometry (BMI), and measures of psychological and physical function [18]. Additionally, the AGES-Reykjavik study also had childhood (age 8–13 years) anthropometric measures from 2120 participants from the 2 main schools in Reykjavik [19]. Data from school records were only available from 1929 and onwards which means that no growth data was available for 8-year olds born before 1921 (i.e. aged over 81 during AGES-Reykjavik study). The childhood anthropometric data was thus gathered for children who were 8–13 during 1929–1947 (Fig. 1).

For this analysis we selected AGES-Reykjavik participants who had childhood anthropometric measurements available at age 8 or 13 years (*n* = 938) and who had BMI data at late-life. An additional 49 individuals were excluded due to missing values on late-life mental health, leaving 889 participants for the main analyses.

### Measures

#### Anthropometric data

Childhood and adolescent growth measurements were extracted from archived school records. Yearly childhood weight and height measurements taken by a trained school nurse were available from ages 8 to 13 years. For this analysis we chose to use the weights and heights from age 8 and 13 years (referred to as childhood weight) as it gives a spread in age. BMI was used as a continuous variable and categorized into normal and overweight/obese. The use of BMI as a categorical variable was primarily because we expected the relationship between BMI and depression to be non-linear. However the use of BMI as a continuous variable has the advantage of providing more statistical power. To make anthropomorphic data at different ages comparable, BMI in childhood/adolescence was translated to BMI at age 18 years using the sex and age (in half year intervals) specific BMI cut-offs of the paper of Cole et al. [20]. Subsequently, BMI categories were made. Initially, three categories were made (normal, thin and overweight/obese) that correspond to underweight (BMI < 18 kg/m^2^) normal weight (BMI ≥18,< 25 kg/m^2^) and overweight or obese (BMI ≥ 25 kg/m^2^) classes at age 18, however as there was no difference between the thin and normal group these two groups were combined. Thus, for a girl aged between 7.5 and 8 years a BMI of 18.03 kg/m^2^ translates to a BMI of 25 kg/m [2] at age 18 years, and is therefore classified as overweight/obese, whereas for a boy of the same age this would be a BMI of 18.16 kg/m^2^. Late-life weight and height were assessed during the AGES-Reykjavik clinical examination (2002–2006). Participants’ height was measured to the nearest 0.5 cm and weight to the nearest 0.1 kg, in subjects without shoes and in light undergarments. BMI was calculated and categorized according to the World Health Organisation (WHO) classifications: < 25 kg/m^2^ (normal/ underweight), 25 to < 30 kg/m^2^ (overweight), ≥ 30 kg/m^2^ (obese).

#### Depression

Late-life depressive symptoms were assessed during the AGES-Reykjavik data collection by using the 15-item version of the Geriatric Depression Scale (GDS) translated into Icelandic [21, 22]. The score was used continuously and was also dichotomized with a score of ≥5 indicating depressive symptoms [23].

The presence of lifetime major depressive disorder (MDD) was assessed at late-life during the AGES-Reykjavik data collection (2002–2006) according to the Diagnostic and Statistical Manual of Mental Disorders (DSM)-IV [24] criteria using the Mini International Neuropsychiatric Interview (MINI) [25]. The MINI is a short diagnostic tool designed to generate a diagnosis for depression and has been validated to yield a reliable DSM-IV diagnosis [26]. To ensure reliable answers, only individuals with no diagnosis of dementia or a score of > 21 on the Mini-mental state examination (MMSE) [27] were eligible to receive the MINI, those who had dementia or a score of > 21 were excluded from the analysis. For efficiency, a preselected group completed the MINI. Individuals were selected if they (i) had a GDS score ≥ 6 *or* (ii) had a GDS score of 4 or 5 and a positive response to 3 out of the 4 following anxiety questions “In the past month, have you felt anxious or frightened?”; “Were there times lately that you felt anxious?”; “Are there special situations that make you anxious?”; “Have you ever had attacks of fear or panic?”, *or* (iii) if they reported ever to have had a doctor diagnosis of depression, *or* (iv) reported previous use of antidepressant medications, *or* (v) were currently using antidepressant medication as evidenced from medication bottles brought to the interview. Based on the MINI, individuals were classified as “ever” versus “never” having MDD to create the variable lifetime MDD. Those classified as ever having MDD were asked at what age they first had symptoms.

#### Covariates

Covariates were assessed at late-life during the AGES-Reykjavik data collection. They were selected a priori based on findings from other studies. We considered level of education attained (primary, secondary, college, university), smoking habits (never, former, current), alcohol consumption (grams/week) and current amount of physical activity (never, rarely, occasionally, moderate, high) as potential confounders.

#### Statistical analysis

For continuous variables the population was described using means and standard deviations (SD); or medians and interquartile ranges for non-normally distributed variables. Percentages were used to describe categorical variables.

Logistic regression analysis was used to estimate the odds ratios for late-life depressive symptoms using the dichotomized GDS score in relation to childhood and adolescent BMI (kg/m^2^) or BMI categories (underweight/normal weight vs. overweight/obesity). Three models were made: the first adjusted for sex and the second additionally included late-life lifestyle factors (education, current physical activity, smoking and alcohol use). The third model additionally included BMI during late-life. Additionally, linear models were made using a continuous logarithmic GDS score (GDS score was not normally distributed) and BMI (kg/m^2^), adjusted for sex. Logistic regression models with adjustments for sex (model 1) and lifestyle variables (model 2) were used to estimate the relationship of BMI at age 8 and 13 years with lifetime MDD (yes/no). In order to eliminate reverse causality, those who had developed MDD before the age of 13 years (*n* = 3) were excluded from the MDD analysis. Missing data among the covariates was small (≤3%) and was therefore ignored (e.g. available case analysis). Participants with no anthropometric data were examined to see if their sociodemographic charicteristics and late-life depressive symptoms significantly differed from those who did have anthropometric data. Analysis were conducted in SPSS version 23 (Inc., Chicago, Illinois, USA) and statistical significance was set at *P* < 0.05.

## Results

A total of 889 individuals who had complete late-life GDS data and childhood BMI information available for either age 8 (*n* = 664) or age 13 (*n* = 711) from school record dating back to1929–1947 were included. Those excluded from this analysis due to lack of childhood/adolescence anthropometric data (i.e. participants from the AGES-Reykjavik study who did not attend one of the two schools in Reykjavik from where the childhood/adolescence data were obtained), had significantly lower late-life GDS scores and were slightly older than those eligible for inclusion. From the included individuals 101 (11.2%) had a late-life GDS ≥5 and 36 (4.1%) had a lifetime MDD with an onset after age 13 years (39 (4.4% after age 8 years). The median GDS score was 2 (interquartile range 1–3). The average self-reported age of MDD onset was 43.5 years (standard deviation (SD) 20.2). Just over half were female and the average age at which they attended the AGES-Reykjavik study measurement was 74.9 years (SD 4.5) (Table 1). Only a few persons were overweight or obese at age 8 or 13 years (*n* = 23, 3.5% and *n* = 26, 3.7%, respectively). During adulthood this number rose markedly, with 68.4% being overweight or obese by late-life.

**Table 1:** Descriptive characteristics of the Reykjavik-AGES sample at late-life (age ~ 75y) with historical anthropometric data

	Total population (*N* = 889)	
Age (years) mean (SD)	74.9	(4.5)
Female n, %	482	54.2
Education n, %		
Primary/secondary	613	68.9
College	155	17.5
University	121	13.6
Smoking status n, %		
Former	424	47.7
Current	124	13.9
Never	341	38.4
Alcohol intake (g/week), median [IQR]	3.2	[0–16]
Physical activity n, %		
Never/rarely	523	58.9
Occasionally/moderate	218	24.5
High	148	16.6
BMI late life (age ~ 75) (kg/m^2^), mean (SD)	27.5	(4.6)
BMI categories late-life n, %^1^		
Normal	278	31.6
Overweight	363	41.3
Obese	238	27.1
GDS score (age ~ 75), median [IQR]	2.1	[1–3]
GDS score ≥ 5 n, %	33	11.2
Lifetime MDD n, %		4.4
BMI age 8 (kg/m^2^), mean (SD)	15.9	(1.2)
BMI categories age 8^1^ n, %		
Normal/Underweight	641	96.5
Overweight/obese	23	3.5
BMI age 13 (kg/m^2^), mean (SD)	18.3	(2.1)
BMI categories age 13^1^ n, %		
Normal/Underweight	685	95.3
Overweight/obese	26	3.7

*BMI* Body Mass Index, *GDS* Geriatric depression scale, *MDD* Major depressive disorder, *SD* standard deviation, *IQR* Interquartile range.

^1^Based on cut-offs from Cole et al., (2000)

### Childhood/adolescent BMI & late- depressive symptoms

BMI at age 8 or 13 years was not associated with having current late-life depressive symptoms using a GDS cut-off of ≥5 (Odds Ratio [OR] 0.99 95% confidence interval [CI] 0.81–1.21 and OR: 0.94 CI: 0.84–1.06 respectively) (Table 2). Similarly, no significant relationships were found in being overweight/obese at age 8 or 13 years (compared to normal BMI) with current late-life depressive symptoms. Adjustment for life-style factors or BMI during late-life did not change these results. Similar results were found when the GDS was used as a continuous score as neither BMI at age 8 or 13 years were significantly associated with the GDS score (β: -0.07 95% CI: − 0.05, 0.03, β: -0.06 95% CI: − 0.03, 0.02 (sex adjusted) respectively (data not shown)).

**Table 2:** Association between childhood BMI with late-life depressive symptoms^1^ in an Icelandic population (*N* = 889)

	Late-life depressive symptoms *(cases/n)*	Model 1^2^			Model 2^3^			Model 3^4^		
		Odds Ratio 95% CI		*P*-value	Odds Ratio 95% CI		*P*-value	Odds Ratio 95% CI		*P*-value
Continuous BMI										
BMI age 8 (kg/m^2^)	69/664	0.99	(0.81–1.21)	0.91	0.98	(0.79–1.20)	0.81	0.97	(0.78–1.20)	0.76
BMI age 13 (kg/m^2^)	84/711	0.94	(0.84–1.06)	0.31	0.95	(0.85–1.07)	0.43	0.95	(0.83–1.07)	0.37
BMI categories										
Normal or underweight age 8	66/641	1.00	(reference)		1.00	(reference)		1.00	(reference)	
Obese or overweight age 8	3/23	1.12	(0.32–3.90)	0.86	0.95	(0.26–3.52)	0.94	0.95	(0.25–3.49)	0.93
Normal or underweight age 13	82/685	1.00	(reference)		1.00	(reference)		1.00	(reference)	
Obese or overweight age 13	2/26	0.59	(0.14–2.53)	0.47	0.61	(0.13–2.72)	0.51	0.59	(0.13–2.65)	0.49

*BMI* Body mass index, *GDS* Geriatric depression scale, *CI* Confidence intervals.

^1^ GDS score ≥ 5 measured at age ~ 75 y

^2^ Model 1 = adjusted for sex

^3^ Model 2 = Model 1 + education, physical activity, smoking status and alcohol use at late-life

^4^ Model 3 = Model 2 + BMI at late-life

### Childhood/adolescent BMI & lifetime MDD

After adjustment for sex, a modest but non-statistically significant association was observed between BMI at age 8 and 13 years and increased risk of lifetime MDD (OR:1.15; CI:0.88–1.50, OR:1.14; CI: 0.98–1.32 respectively) (Table 3). Being overweight or obese at age 8 was associated with increased risk of lifetime MDD (OR: 4.30; CI: 1.34–13.76) when compared to having a normal BMI. Although the odds ratio of lifetime MDD was also elevated for being overweight or obese at age 13 years (OR = 3.00) this did not reach statistical significance. Adjustment for late-life lifestyle factors slightly attenuated the odds ratios (e.g. OR for lifetime MDD 4.03 CI 1.16–13.96 for overweight or obesity at age 8 compared to normal BMI).

**Table 3:** Association between childhood BMI with lifetime MDD^1^ in an Icelandic population (*N* = 889)

	Lifetime MDD *(cases/n)*	Model 1^2^			Model 2^3^		
		Odds Ratio 95% CI		*P*-value	Odds Ratio 95% CI		*P*-value
Continuous BMI							
BMI age 8 (kg/m^2^)	32/664	1.15	(0.88–1.50)	0.32	1.11	(0.84–1.47)	0.46
BMI age 13 (kg/m^2^)	30/708	1.14	(0.98–1.32)	0.10	1.12	(0.96–1.32)	0.14
BMI categories							
Normal or underweight age 8	24/641	1.00	(reference)		1.00	(reference)	
Obese or overweight age 8	4/23	4.30^4^	(1.34–13.76)	0.01	4.03^4^	(1.16–13.96)	0.03
Normal or underweight age 13	27/683	1.00	(reference)		1.00	(reference)	
Obese or overweight age 13	3/25	3.00	(0.84–10.73)	0.09	2.65	(0.69–10.26)	0.16

*BMI* Body mass index, *MDD* Major Depressive Disorder, *GDS* Geriatric depression scale, *CI* Confidence intervals.

^1^ measured at age ~ 75 y

^2^ Model 1 = adjusted for sex

^3^ Model 2 = Model 1 + education, physical activity, smoking status and alcohol use

^4^ Significant at *P*-value <0.05

## Discussion

This study reports on measured childhood/adolescence BMI with follow-up depression data more than 60 years later. Our findings show that being overweight or obese during childhood/adolescence is not associated with depressive symptoms during late-life. However, being overweight at age 8 (and possibly age 13 years) was associated with a significant increased risk of lifetime MDD. However, our results must be taken with caution due to the low prevalence of overweight/obese at young age and the low prevalence of participants with lifetime MDD in this cohort.

Only one other study has compared childhood/adolescent overweight/obesity with depressive symptoms during late-life in 4410 participants. The study found that women who were overweight in adolescence were significantly more likely to experience depressive symptoms at age 65 than their normal weight counterparts, although no relationship was observed for men [6]. This was not confirmed in our results, as we found no associations between childhood and adolescent obesity and late-life depressive symptoms. Differences between the two studies may be that we used measured childhood weight and height to obtain BMI, and the comparative study used a relative BMI based on high school photos (age 14–18). Also, the age at which BMI was measured in our study was slightly younger. Further, our small sample size, preventing us from performing an analysis stratified by sex, could also explain the differences. Another important point is that we assessed late-life depression at age 66–86, which is on average 10 years older than the comparative study, increasing the risk of other important factors which may contribute to current depressive symptoms such as chronic disease, frailty, poor physical functioning and sleep disturbance [28, 29].

Our study found that being overweight at age 8 and 13 is associated with an increased risk of lifetime MDD, although only the odds for age 8 reach statistical significance. Comparison to other studies is difficult as the age ranges and follow-up durations used are varied. Three other studies found significant associations between childhood/adolescence obesity (measured at ages 9–18 years, 5 years and 7–15 years, respectively) and a DSM based diagnosis of depression 20–30 years later [9, 14, 30]. However, in one of these studies statistical significance was only apparent in females but not in males. Interestingly, studies that do not take a lifetime approach, i.e. with very short follow-up periods, tend to find no relationship between childhood or adolescent obesity (ages 11–17) and subsequent MDD [13, 31]. The lack of significant associations between childhood obesity and MDD onset in these studies could be explained by the very short follow-up periods (1–4 years). A meta-analysis has also observed that stronger associations between adolescence obesity and depression were found with longer follow-up periods (more than 10 years) [8]. It may be that the duration of the exposure to obesity is of relevance to the development of depression or that a longer time period is required for childhood obesity to have an effect on a psychiatric diagnosis. Our lack of findings between overweight and obesity at age 13 with lifetime MDD is most likely due to the insufficient numbers of obese/overweight 13 year olds developing MDD. Our cohort had a particularly low prevalence of overweight/obese children (3.7% at age 13).

Our study focuses on the critical period of childhood when the relationship between obesity and depression may develop. This relationship is complex and many mechanisms have been proposed. One of the most widely proposed mechanism linking childhood obesity to subsequent depression is low self-esteem which is frequently observed in those who do not conform to the cultural ideal body weight [32]. Low self-esteem has been associated with subsequent depression [10]. Furthermore, overweight children are more frequently subjected to bullying which can also lead to increased stress [33]. The impact of body dissatisfaction on self-esteem during adulthood could be less than in younger ages, and adult bullying is also less common. Another possibility is that the shared vulnerability for both overweight and depression is due in part to a shared genetic risk [12]. One study indicated that 12% of the genetic component of depression is shared with obesity [34], and an even more recent genome-wide association study has suggested genetic risk for MDD is correlated with body mass [35]. Furthermore, it has been suggested that physical inactivity and an unhealthy diet may not only impact depression via obesity but that an unhealthy lifestyle may have an additive effect over and above the obese status [36].

Alternatively, metabolic dysregulation resulting from the cumulative long term exposure of an unhealthy BMI could partly explain the association between BMI and depression. Inflammation is a factor common to both obesity and depression, although it has been suggested that obesity and inflammation are outcomes of adolescent depression, rather than contributing causes [37]. Alternatively, resistance to leptin may constitute a risk for depression. Leptin is a hormone produced in proportion to fat mass which controls appetite and energy expenditure. Leptin also has an impact on mood. Animal models have shown that peripheral and central administration of leptin produces antidepressant-like effects. Leptin resistance, a characteristic of severe obesity (BMI ≥ 35 kg/m2), due to impaired leptin transport across the blood–brain barrier, reduces the function of leptin receptors, and defects in leptin signal transduction [38]. Finally, overweight and obesity over the long term are risk factors for somatic diseases which themselves are associated with poorer mental health. There is no simple pathway from body weight to depression. Most likely, a combination of factors will play a role.

The strengths of this study are the long-term follow-up enabling us to adopt a life-course approach to weight and depression. We used measured height and weight, also at childhood age, and we had two different measures of depression, depressive symptoms at late-life and a clinical diagnosis of past depression, both measured late-life. However, there are also some limitations. The main limitation was the low prevalence of overweight/obesity (3.5% at age 8, 3.7% at age 13 years) and lifetime MDD (4.4%). Current Icelandic statistics on obesity show that 23% are overweight at age 9, and 22% are overweight or obese at age 13 years [39]. The low prevalence of childhood overweight and obesity is partly a result of birth cohort differences. During the 1920’s-40’s overweight and obesity would more likely be a result of genetic vulnerability than environmental influences [40]. The low prevalence of lifetime MDD compared to current estimates of 15–25% [41] has previously been noted in this cohort [42]. The prevalence of MDD and current depressive symptoms may be lower because current depression is a risk factor for non-response and for earlier mortality. Furthermore those with a MMSE score < 21 were excluded, and given that depression and dementia/mild cognitive impairment are highly comorbid [43], there is an increased likelihood that depressed persons were excluded. The low number of participants having lifetime MDD is partly reflective of the era into which they were born. Unlike in the majority of European countries and in Northern America, depression was not given much attention in Iceland until the 1980’s by which time these participants would already be middle aged. However, assuming childhood overweight is associated with MDD, the low prevalence of childhood overweight may partly explain the low prevalence of MDD. The consequence of such a low prevalence means that this study was poorly powered and the risk estimates could be inflated. However, as the findings are biologically plausible and for the most part confirmed by other studies, we assume the general direction of association to be true. Additionally, there were insufficient data to explore the previously reported modifying effect of sex for MDD or whether childhood overweight/obesity was related to an earlier onset of MDD. Another limitation is that we had no data on change in covariates or childhood covariates, such as parental education of social economic status, the latter of which is associated with both depression and BMI. Examining life-long MDD retrospectively from age 75 may be limited by the fact that the recall period is long. Finally, this study could be subject to a selection bias (those with poor health such as high obesity and depression) may not survive up to age 75, or to increased rates of non-response, which may have caused an underestimation of the true associations.

## Conclusion

Within this Icelandic sample, being overweight/obese during childhood is associated with lifetime MDD, but no associations were observed with late-life depressive symptoms. The low prevalence of childhood overweight in our data reflects the time period the study was conducted. Given that more adolescents are obese today than previously, understanding the mechanisms of the associations between childhood obesity and depression in later life will be of great importance. Our research implies that childhood weight is an important determinant of subsequent adult mental health and therefore studies examining childhood obesity and lifetime MDD in populations where childhood obesity is more prevalent are warranted.

## Data Availability

The data that support the findings of this study are available from Icelandic Heart Association but restrictions apply to the availability of these data, which were used under license for the current study, and so are not publicly available. Data are however available from the authors upon reasonable request and with permission of Icelandic Heart Association.

## Abbreviations

AGES: Age, Gene/Environment Susceptibility
BMI: Body mass index
CI: Confidence intervals
DSMI: Diagnostic and Statistical Manual of Mental Disorders
GDS: Geriatric depression scale
MDD: Major depressive disorder
MINI: Mini International Neuropsychiatric Interview
MMSE: Mini-mental state examination
OR: Odds ratio
SD: Standard deviation
WHO: World Health Organisation

## References

[1] Ng M, Fleming T, Robinson M, Thomson B, Graetz N, Margono C (2014). Global, regional, and national prevalence of overweight and obesity in children and adults during 1980–2013: a systematic analysis for the global burden of disease study 2013. Lancet.

[2] Okasha M, McCarron P, McEwen J, Smith GD (2002). Body mass index in young adulthood and cancer mortality: a retrospective cohort study. J Epidemiol Community Health.

[3] Llewellyn A, Simmonds M, Owen CG, Woolacott N (2016). Childhood obesity as a predictor of morbidity in adulthood: a systematic review and meta-analysis. Obes Rev.

[4] Huxley RR, Shiell AW, Law CM (2000). The role of size at birth and postnatal catch-up growth in determining systolic blood pressure: a systematic review of the literature. J Hypertens.

[5] Imai CM, Gunnarsdottir I, Gudnason V, Aspelund T, Birgisdottir BE, Thorsdottir I (2014). Faster increase in body mass index between ages 8 and 13 is associated with risk factors for cardiovascular morbidity and mortality. Nutr Metab Cardiovasc Dis.

[6] Martinson ML, Vasunilashorn SM (2016). The long-arm of adolescent weight status on later life depressive symptoms. Age Ageing.

[7] Mühlig Y, Antel J, Föcker M, Hebebrand J (2016). Are bidirectional associations of obesity and depression already apparent in childhood and adolescence as based on high-quality studies? A systematic review. Obes Rev.

[8] Mannan M, Mamun A, Doi S, Clavarino A (2016). Prospective associations between depression and obesity for adolescent males and females- a systematic review and meta-analysis of longitudinal studies. PLoS One.

[9] Sanderson K, Patton GC, McKercher C, Dwyer T, Venn AJ (2011). Overweight and obesity in childhood and risk of mental disorder: a 20-year cohort study. Aust N Z J Psychiatry.

[10] Rieger S, Göllner R, Trautwein U, Roberts BW (2016). Low self-esteem prospectively predicts depression in the transition to young adulthood: a replication of Orth, robins, and Roberts (2008). J Pers Soc Psychol.

[11] Jackson KL, Janssen I, Appelhans BM, Kazlauskaite R, Karavolos K, Dugan SA (2014). Body image satisfaction and depression in midlife women: the study of Women’s health across the nation (SWAN). Arch Womens Ment Health.

[12] Samaan Z, Lee YK, Gerstein HC, Engert JC, Bosch J, Mohan V (2015). Obesity genes and risk of major depressive disorder in a multiethnic population. J Clin Psychiatry.

[13] Roberts RE, Duong HT (2013). Obese youths are not more likely to become depressed, but depressed youths are more likely to become obese. Psychol Med.

[14] Sánchez-Villegas A, Pimenta AM, Beunza JJ, Guillen-Grima F, Toledo E, Martinez-Gonzalez MA (2010). Childhood and young adult overweight/obesity and incidence of depression in the SUN project. Obesity (Silver Spring).

[15] Viner Russell M, Cole Tim J (2005). Adult socioeconomic, educational, social, and psychological outcomes of childhood obesity: a national birth cohort study. BMJ.

[16] Geoffroy MC, Li L, Power C (2014). Depressive symptoms and body mass index: co-morbidity and direction of association in a British birth cohort followed over 50 years. Psychol Med.

[17] Guo SS, Chumlea WC (1999). Tracking of body mass index in children in relation to overweight in adulthood. Am J Clin Nutr.

[18] Harris TB, Launer LJ, Eiriksdottir G, Kjartansson O, Jonsson PV, Sigurdsson G (2007). Age, gene/environment susceptibility-Reykjavik study: multidisciplinary applied phenomics. Am J Epidemiol.

[19] Halldorsson TI, Gunnarsdottir I, Birgisdottir BE, Gudnason V, Aspelund T, Thorsdottir I (2011). Childhood growth and adult hypertension in a population of high birth weight. Hypertension.

[20] Cole TJ, Bellizzi MC, Flegal KM, Dietz WH (2000). Establishing a standard definition for child overweight and obesity worldwide: international survey. BMJ.

[21] Yesavage Jerome A., Brink T.L., Rose Terence L., Lum Owen, Huang Virginia, Adey Michael, Leirer Von Otto (1982). Development and validation of a geriatric depression screening scale: A preliminary report. Journal of Psychiatric Research.

[22] Valdimarsdóttir M, Jónsson JE, Einarsdóttir S, Tómasson K (2000). Validation of an Icelandic version of the geriatric depression scale (GDS). Laeknabladid.

[23] Almeida OP, Almeida SA (1999). Short versions of the geriatric depression scale: a study of their validity for the diagnosis of a major depressive episode according to ICD-10 and DSM-IV. Int J Geriatr Psychiatry.

[24] American Psychiatric Association (APA). Diagnostic and statistical manual of mental disorders. 4th ed. Washington DC: American Psychiatric Association; 1996.

[25] Sheehan D V, Lecrubier Y, Sheehan KH, Amorim P, Janavs J, Weiller E, et al. The Mini-International Neuropsychiatric Interview (M.I.N.I.): the development and validation of a structured diagnostic psychiatric interview for DSM-IV and ICD-10. J Clin Psychiatry. 1998;22–33;quiz 34–57 [cited 2017 22 Feb]. 9881538

[26] Amorim P, Lecrubier Y, Weiller E, Hergueta T, Sheehan D. DSM-IH-R Psychotic Disorders: procedural validity of the Mini International Neuropsychiatric Interview (MINI). Concordance and causes for discordance with the CIDI. Eur Psychiatry. 1998 Jan;13(1):26–34 [cited 2018 25 Jun]. 10.1016/S0924-9338(97)86748-X 19698595

[27] Folstein MF, Folstein SE, McHugh PR (1975). “Mini-mental state”;. A practical method for grading the cognitive state of patients for the clinician. J Psychiatr Res.

[28] Cole MG, Dendukuri N (2003). Risk factors for depression among elderly community subjects: a systematic review and meta-analysis. Am J Psychiatry.

[29] Makizako Hyuma, Shimada Hiroyuki, Doi Takehiko, Yoshida Daisuke, Anan Yuya, Tsutsumimoto Kota, Uemura Kazuki, Liu-Ambrose Teresa, Park Hyuntae, Lee Sanyoon, Suzuki Takao (2015). Physical Frailty Predicts Incident Depressive Symptoms in Elderly People: Prospective Findings From the Obu Study of Health Promotion for the Elderly. Journal of the American Medical Directors Association.

[30] Anderson SE, Cohen P, Naumova EN, Jacques PF, Must A (2007). Adolescent obesity and risk for subsequent major depressive disorder and anxiety disorder: prospective evidence. Psychosom Med.

[31] Boutelle H, Fulkerson CS (2010). Obesity as a prospective predictor of depression in adolescent females. Health Psychol.

[32] Wang F, Wild TC, Kipp W, Kuhle S, Veugelers PJ (2009). The influence of childhood obesity on the development of self-esteem. Heal Rep.

[33] van Geel M, Vedder P, Tanilon J (2014). Are overweight and obese youths more often bullied by their peers? A meta-analysis on the relation between weight status and bullying. Int J Obes.

[34] Afari N, Noonan C, Goldberg J, Roy-Byrne P, Schur E, Golnari G (2010). Depression and obesity: do shared genes explain the relationship?. Depress Anxiety.

[35] Wray NR, Sullivan PF (2017). Genome-wide association analyses identify 44 risk variants and refine the genetic architecture of major depression. BioRxiv.

[36] Yu ZM, Parker L, Dummer TJB (2014). Depressive symptoms, diet quality, physical activity, and body composition among populations in Nova Scotia, Canada: report from the Atlantic Partnership for Tomorrow’s health. Prev Med (Baltim).

[37] Byrne ML, O’Brien-Simpson NM, Mitchell SA, Allen NB (2015). Adolescent-onset depression: are obesity and inflammation developmental mechanisms or outcomes?. Child Psychiatry Hum Dev.

[38] Milaneschi Yuri, Simmons W. Kyle, van Rossum Elisabeth F. C., Penninx Brenda WJH (2018). Depression and obesity: evidence of shared biological mechanisms. Molecular Psychiatry.

[39] Jónsson S, Hedinsdottir M, Erlendsdottir RO, Gudlaugsson J, Danielsdottir S, Reynisson J. Líkamsþyngd barna á höfuðborgarsvæðinu (body weight of children in the metropolitan area. Reykjavík Embætti landlæknis og Heilsugæsla höfuðborgarsvæðisins 2013;8–11.

[40] Hill JO, Peters JC (1998). Environmental contributions to the obesity epidemic. Science.

[41] Vilhelmsson A (2013). Depression and antidepressants: a Nordic perspective. Front Public Heal.

[42] Geerlings M. I., Sigurdsson S., Eiriksdottir G., Garcia M. E., Harris T. B., Sigurdsson T., Gudnason V., Launer L. J. (2012). Associations of current and remitted major depressive disorder with brain atrophy: the AGES–Reykjavik Study. Psychological Medicine.

[43] Thomas AJ, OʼBrien JT. (2008). Depression and cognition in older adults. Curr Opin Psychiatry.

